# Targeted Next-Generation Sequencing of Cancer-Related Genes in a Norwegian Patient Cohort With Head and Neck Squamous Cell Carcinoma Reveals Novel Actionable Mutations and Correlations With Pathological Parameters

**DOI:** 10.3389/fonc.2021.734134

**Published:** 2021-09-24

**Authors:** Harsh N. Dongre, Hilde Haave, Siren Fromreide, Fredrik A. Erland, Svein Erik Emblem Moe, Sophia Manueldas Dhayalan, Rasmus Kopperud Riis, Dipak Sapkota, Daniela Elena Costea, Hans Jorgen Aarstad, Olav K. Vintermyr

**Affiliations:** ^1^ Department of Pathology, Haukeland University Hospital, Bergen, Norway; ^2^ Gade Laboratory for Pathology, Department of Clinical Medicine, University of Bergen, Bergen, Norway; ^3^ Centre for Cancer Biomarkers CCBIO, Department of Clinical Medicine, University of Bergen, Bergen, Norway; ^4^ Department of Otolaryngology/Head and Neck Surgery, Haukeland University Hospital, Bergen, Norway; ^5^ Otolaryngology, Department of Clinical Medicine, Faculty of Medicine, University of Bergen, Bergen, Norway; ^6^ Institute of Oral Biology, Faculty of Dentistry, University of Oslo, Oslo, Norway

**Keywords:** next-generation sequencing (NGS), actionable mutation, pathological parameters, stromal desmoplasia, inflammation, head and neck squamous cell carcinoma (HNSCC)

## Abstract

**Background:**

Targeted next-generation sequencing (NGS) is increasingly applied in clinical oncology to advance personalized treatment. Despite success in many other tumour types, use of targeted NGS panels for assisting diagnosis and treatment of head and neck squamous cell carcinomas (HNSCC) is still limited.

**Aim:**

The focus of this study was to establish a robust NGS panel targeting most frequent cancer mutations in long-term preserved formalin-fixed paraffin-embedded (FFPE) tissue samples of HNSCC from routine diagnostics.

**Materials and Methods:**

Tumour DNA obtained from archival FFPE tissue blocks of HNSCC patients treated at Haukeland University Hospital between 2003-2016 (n=111) was subjected to mutational analysis using a custom made AmpliSeq Library PLUS panel targeting 31 genes (Illumina). Associations between mutational burden and clinical and pathological parameters were investigated. Mutation and corresponding clinicopathological data from HNSCC were extracted for selected genes from the Cancer Genome Atlas (TCGA) and used for Chi-square and Kaplan-Meier analysis.

**Results:**

The threshold for sufficient number of reads was attained in 104 (93.7%) cases. Although the specific number of PCR amplified reads detected decreased, the number of NGS-annotated mutations did not significantly change with increased tissue preservation time. In HPV-negative carcinomas, mutations were detected mainly in *TP53* (73.3%), *FAT1* (26.7%) and *FLG* (16.7%) whereas in HPV-positive, the common mutations were in *FLG (24.3%) FAT1* (17%) and *FGFR3* (14.6%) genes. Other less common pathogenic mutations, including well reported SNPs were reproducibly identified. Presence of at least one cancer-specific mutations was found to be positively associated with an extensive desmoplastic stroma (p=0.019), and an aggressive type of invasive front (p=0.035), and negatively associated with the degree of differentiation (p=0.041). Analysis of TCGA data corroborated the association between cancer-specific mutations and tumour differentiation and survival analysis showed that tumours with at least one mutation had shorter disease-free and overall survival (p=0.005).

**Conclusions:**

A custom made targeted NGS panel could reliably detect several specific mutations in archival samples of HNSCCs preserved up to 17 years. Using this method novel associations between mutational burden and clinical and pathological parameters were detected and actionable mutations in HPV-positive HNSCC were discovered.

## Introduction

Head and neck squamous cell carcinoma (HNSCC), arising in the oral cavity, oropharynx, hypopharynx and larynx, is a major public health concern worldwide (1). The incidence and mortality rates of HNSCC are increasing, with approximately 750,000 new cases and 360,000 deaths reported in 2020 (1). HNSCC are characterized by aetiological, phenotypical, biological, and clinical heterogeneity (2, 3). Smoking and smokeless tobacco chewing habits in developing countries and human papillomavirus (HPV) infection in developed countries are emerging as important risk factors for the rise in incidence rates of HNSCC (2, 3). Given the reported differences in survival outcomes based on HPV and smoking status, establishing HPV as a prognostic factor for better survival in oropharyngeal SCC (OPSCC) (4, 5), there is an additional interest in understanding the molecular differences between HPV-positive and HPV-negative lesions especially in OPSCC.

Current cancer treatment is moving towards more personalized and targeted treatment. Both targeted and immunotherapies have improved 5-year survival rates significantly in many types of cancers (6–8). However, for patients with HNSCC the only targeted therapy approved is cetuximab, a monoclonal antibody against epidermal growth factor receptor (EGFR) (6, 9). Recently, pembrolizumab and nivolumab, immunotherapeutic agents, have been approved for HNSCC (10, 11). Nonetheless, patients treated with targeted therapy such as cetuximab combined with radiotherapy develop resistance (6, 12) and less than 20% of HNSCC patients treated with immunotherapy achieve a lasting response (13).

Several studies have investigated genomic aberrations associated with HNSCC and this has greatly increased our understanding of the mutational landscape of HNSCC (2, 3, 14–16). Moreover, with the advent of next-generation sequencing (NGS), several studies have added to the ever-growing list of novel genetic alterations in HNSCC. Frequent mutations in several genes including *TP53, CDKN2A, PIK3CA, NOTCH1, CASP8* and *MLL2 (KMT2D)* among others as well as alterations in *EGFR, CCND1* and *FGFR* have been reported (14–16). However, these genes are yet to translate into clinically beneficial prognostic or predictive biomarkers. The incorporation of NGS into routine clinical setting has been hampered by lack of large number of biopsy specimens. Formalin-fixed paraffin-embedded (FFPE) archival tissues could overcome this as they are a rich resource that could allow correlations of specific mutational landscape with clinical and pathological parameters in long-term follow-up retrospective studies.

The aim of this study was to establish a robust NGS panel targeting most frequent cancer mutations in long-term preserved FFPE samples of HNSCC that would allow testing of clinical outcome and correlations with clinical and pathological parameters of cancer-specific mutations. This method could serve for identification of novel, actionable mutations that would open new avenues for personalized therapy in HNSCC. In addition, it will also bring new insights into the biology of this disease by allowing investigation of correlations between mutational landscape of tumours, clinical variables, and histopathological parameters, which is difficult to assess in fresh-frozen tissues. In this study we report that a custom made targeted NGS panel could reliably detect several specific mutations in archival samples of HNSCCs preserved up to 17 years. Using this method, we could identify targetable mutations in HNSCC and reveal novel associations between the mutational load and clinicopathological variables like tumour desmoplasia, pattern of invasion at the tumour front and the degree of differentiation.

## Materials and Methods

### Patient Cohort

The cohort consisted of consecutive patients diagnosed with HNSCC at the Department of Otolaryngology/Head & Neck Surgery, Haukeland University Hospital (HUH), Bergen, Norway between 2003 to 2010 and 2013 to 2016, who consented to participate in the study. In total, 111 HNSCC patients and 9 cancer-free control patients were enrolled in this retrospective study. Due to insufficient number of reads, seven HNSCC patients and two cancer-free controls were excluded from the final analysis. Out of 104 patients with NGS data that passed the threshold of reads, 43 (41.3%) patients were HPV-positive, 57 (54.8%) patients were HPV-negative, and 4 (3.8%) were not tested for HPV. Clinical data (age, gender, smoking, TNM staging at the point of diagnosis according to the American Joint Committee on Cancer (AJCC) manual 6^th^ edition) was obtained from the Electronic Patient Journal at Haukeland University Hospital (DIPS). To ensure continuity in the study with respect to HNSCC classification during the whole period (2003-2016), staging of all the tumours has been set based on the AJCC 6^th^ edition. The study was approved by the Regional (REK Vest) Committee for Medical and Health Research Ethics (2011/125).

### Histopathological Evaluation of Haematoxylin and Eosin (HE) Sections

All morphological evaluations were done on representative HE sections from FFPE blocks by an expert pathologist (OKV) and a specialist in surgery (HH). The morphological scoring was done by consensus evaluation based on a pre-defined list of criteria and without knowledge of the HPV status following the scoring system described elsewhere (17, 18). The following tumour phenotypic criteria were evaluated: a) degree of keratinization, b) fraction maturating cells (%), and c) tumour stromal invasion pattern. In addition, the following host response patterns to tumour were evaluated: d) inflammatory response and e) stromal desmoplasia. Each parameter was scored 1-4 and further defined in **Supplementary Table 1** in detail. Degree of keratinization was defined as tumour area with apparent keratinized single cells, groups of cells or areas with remnants of keratinized cells. Fraction maturing cells was denoted as the fraction of immature (basal like cells) cells relative to the more differentiated cell fraction in the tumour sample. The invasion pattern was evaluated at the interphase between tumour and the intervening stroma at the invasive tumour front (19). The inflammatory host response was evaluated as the extent of chronic inflammation denoted by the presence of lymphocytes, plasma cells and histiocytic cells surrounding the invading tumour front. Areas with neutrophil granulocytic responses were not considered as these areas may represent secondary host responses due to tumour cell death, infection or ulcerations. Tumour stromal desmoplasia was evaluated only in the context of fibrillary and myxomatous tumour host responses *i.e.*, the angiogenetic tumour host responses was not included in this parameter.

### DNA Isolation and HPV Detection

All tumour samples were reviewed, and representative tissue samples were selected. DNA was extracted from FFPE tissue blocks. These were tissues from primary HNSCC from diagnostic or surgical samples collected 5 to 17 years ago. For the NGS analysis at least 20% tumour fraction was required to be included in the analysis. Many of FFPE blocks were macro-dissected to enrich the tumour fraction of the patient samples. The mean tumour fraction was 47.5% (median 50%) in the samples that were analysed with NGS. Three to six 10 µm thick FFPE sections were de-paraffinized in deparaffinization solution and digested overnight in ATL-buffer and Proteinase K (Qiagen GmbH, Hilden, Germany) at 56°C. DNA was extracted using the E.Z.N.A tissue DNA kit (Omega BioTek, Norcross, GA, USA), and the DNA concentration was quantified using Qubit dsDNA BR assay kit (Thermo Fisher Scientific, Waltham, MA, USA) according to manufacturer’s protocol.

HPV DNA detection was performed using standard Gp5+/Gp6+ primers as described elsewhere (20, 21). p16 status of all HPV-positive OPSCC from 2003-2010 (75% of cohort) was also determined by performing immunohistochemistry (IHC) using anti-p16^INK4a^ monoclonal antibody (clone E6H4, Roche diagnostics, Switzerland) as described elsewhere (5, 22). The cut-off percentage for p16 positivity was set as 70% as described in literature for OPSCC (5).

### Design of Custom Made NGS Panel

The custom made NGS panel was based on previously reported mutational burden in HPV-negative and HPV-positive HNSCC (15, 23, 24). For some genes, the specific targeted regions were adapted from the commercially available Illumina TruSight Tumor 15 (*TP53, NRAS*) and TruSight Tumor 26 (*AKT 1, BRAF, CTNNB1, KIT, PTEN*) NGS panels (Illumina, San Diego, CA, USA). The PCR primer sets for the other targeted gene regions were *de novo* annotated and designed by Illumina DesignStudio. A custom-made NGS panel, targeting hotspot gene exon regions in 28 genes and complete exon coverage in 3 genes (*TP53, TRAF 3 and FAT1*) was designed. The list of genes is shown in **Supplementary Table 2**. Year- and side-matched normal samples (n=7), samples with known specific mutations, and Acrometrix oncology hotspot control (Thermo Fisher Scientific, Waltham, CA, USA) were used for quality control of the NGS panel.

### NGS Library Preparation and Sequencing

DNA library preparation was conducted using the AmpliSeq Library PLUS kit (Illumina, San Diego, CA, USA) according to manufacturer’s instructions. Briefly, 50 ng of DNA was used to prepare amplicon libraries with two custom-made primer pools. The amplicons were then partially digested, ligated to AmpliSeq CD indexes and purified with AMPure XP beads (Beckman Coulter, High Wycombe, UK). Further, the library was again amplified, purified with beads, and then quantified using Qubit dsDNA HS kit (Thermo Fisher Scientific, Waltham, MA, USA). Samples were pooled in equimolar concentrations, and finally 1.3 pM of library solution was loaded for paired-end sequencing on the Illumina MiniSeq platform using the BaseSpace Sequence Hub (Illumina, San Diego, CA, USA).

### Bioinformatics

Bioinformatical analysis was performed using the DNA amplicon v.2.1.1 workflow in BaseSpace (Illumina, San Diego, CA, USA). The targeted regions (specified in a custom manifest file) were aligned to the reference genome hg19/GRCh37 using the Burrows Wheeler Aligner. Variants were called using the Somatic variant caller and annotated by RefSeq using National Centre for Biotechnology Information (NCBI) database. An amplicon total depth (coverage) of more than 500 reads and a variant allele frequency (VAF) of more than 5% were set as strict thresholds to be included in the analysis. The recorded variants were evaluated in dbSNP (National Centre for Biotechnology Information) and the Cosmic (Catalog of Somatic Mutations in Cancer) databases and only coding sequences were considered (25). The European population frequencies for the variants were found in the ALFA Allele Frequency Aggregator (26). If a mutation had an allele frequency of more than 1% it was considered a single nucleotide polymorphism (SNP). Mutations with an allele frequency in the range 0.1% - 1% were also considered a SNP unless they had a pathogenic score of more than 0.5 in the Cosmic database. All mutations recorded were of the non-synonymous type, but for SNPs synonymous mutations were also recorded separately. Four normal FFPE tonsillar tissue samples from the period 2003-2010 and three normal FFPE tonsillar samples from the period 2012-2015 were analysed to validate mutations with respect to unspecific variants due to very aged FFPE tumour samples in this study.

Graphs related to mutational signatures were created using a web based application: Mutational Signatures in Cancer (MuSiCa) (27). Pictorial distribution of SNVs on protein domain structures was plotted using cBioPortal online tool (28, 29).

### Statistical Analysis

The statistical analysis was performed using IBM SPSS Statistics Version 25.0 (Armonk, IBM Corp, USA). The data is presented as mean ± SEM (standard error over mean) and significance set at p<0.05. Tests of independence of clinicopathological parameters with mutational burden were assessed using Pearson’s chi-squared test and Phi correlation coefficient was calculated to measure the strength of association between variables tested. For correlation analysis, cases were categorized into higher and lower mutational burden groups by mean value or a pre-set cut-off point; pathological parameters were dichotomized in lower (1&2) and higher scores (3&4).

### Analysis of TCGA Data

Mutation status for *TP53, FAT1, FLG, CDKN2A, FGFR3, NSD-1*, and *KMT2C* genes and corresponding clinicopathological data for HNSCC (n=496) specimens were exported using cBioPortal online tool (28, 29). The data were imported to IBM SPSS for further statistical analysis. HNSCC were stratified with respect to the mutational status of the examined genes and associations between the mutational status and clinicopathological parameters and 5-year overall survival or 5-year disease (relapse) free survival were examined using Chi-square test, and Kaplan-Meier plot (Log Rank test), respectively.

## Results

### Clinical Characteristics of the HNSCC Cohort

The NGS data from the HNSCC cohort after quality control consisted of 104 patients with SCC at different anatomical head and neck sites: oropharynx (61%), oral cavity (32%), hypopharynx (3%), larynx (2%), and metastatic lymph nodes (3%). The majority of cohort consisted of males (79%), the median age was 63 years (range 38-87), and the majority (68%) had a history of smoking. At the time of diagnosis, 61.5% presented with T1&T2 while 35.6% were at late T3&T4 stages, 54.8% had loco-regional lymph node metastasis, and 3% had distant metastasis.

For determining the HPV status, both HPV DNA and IHC for p16 were performed. The sensitivity and specificity of p16 IHC as compared to HPV DNA detection was 96% and 85.7%, respectively. From all HNSCC cases, 43 (41.3%) were HPV-positive as determined by presence of HPV DNA, with the majority being oropharyngeal SCC (95.4%) and only 2% of oral SCC being HPV-positive. Most of the HPV-positive cancers presented with lymph node metastasis (79.1%) at diagnosis, and only 38.6% of the HPV-negative. A positive moderate correlation between HPV status and locoregional lymph node metastasis was detected (p<0.001, r=0.422). In addition, among the oropharyngeal cancers, 41 out of 63 (66.7%) were HPV-positive while the rest were HPV-negative (33.3%). Out of these 41 HPV-positive cases, 24 cases (58.5) % were T1 and T2 early-stage tumours and 33 cases (80.5%) had lymph node metastasis (p=0.018, r=0.30). Clinical details of all the patients are presented in **Table 1**.

**Table 1:** Clinical parameters of patients.

Characteristics	All patients n = 104 (%)	HPV-positive n = 43 (%)	HPV-negative n = 57 (%)	p-value
Age at diagnosis	63	59	64	0.381
Median	(38-87)	(38-87)	(39-84)	
Sex:				0.230
Male	82 (78.8)	36 (83.7)	42 (73.7)	
Female	22 (21.2)	07 (16.3)	15 (26.3)	
Tumour site:				0.0001*
Oral cavity	33 (31.7)	01 (2.3)	32 (56.1)	
Oropharynx	63 (60.6)	41 (95.4)	22 (38.7)	
Hypopharynx	03 (2.9)	01 (2.3)	01 (1.7)^$^	
Larynx	02 (1.9)	00	02 (3.5)	
Metastasis	03 (2.9)	–	–	
Smoking:				0.137
Yes	71 (68.3)	25 (58.1)	42 (73.7)	
No	33 (31.7)	18 (41.9)	15 (26.3)	
T stage:				0.289
T1 and T2	64 (61.5)	25 (58.1)	39 (68.4)	
T3 and T4	37 (35.6)	18 (41.9)	19 (31.6)	
Unknown	03 (2.9)	–	–	
N stage:				**0.0001**
N0	44 (42.3)	9 (20.9)	35 (61.4)	
N+	57 (54.8)	34 (79.1)	23 (38.6)	
Unknown	03 (2.9)	–	–	
M stage:				0.127
M0	98 (94.2)	43 (100)	55 (94.7)	
M+	03 (2.9)	0	03 (5.3)	
Unknown	03 (2.9)	–	–	
Stage grouping^#^				–
Stage I	12 (11.5)	01 (2.3)	10 (17.5)	
Stage II	19 (18.3)	03 (6.9)	16 (28.1)	
Stage III	11 (10.6)	05 (11.7)	06 (10.6)	
Stage IV	59 (56.7)	34 (79.1)	25 (43.8)	
Unknown	03 (2.9)			

p-value is based on Chi-square test.

*Correlation between oral cavity and oropharynx based on HPV status. ^#^Based on AJCC 6^th^ edition. ^$^HPV DNA test was inconclusive for one patient. Significant values are denoted in bold.

### Clinico-Pathological Correlations

**Figure 1:**
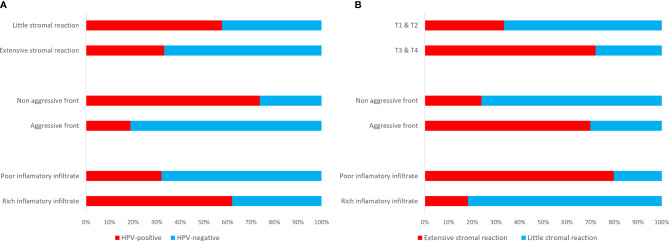
Clinico-pathological correlations depicting an extensive (scores 3&4) stromal reaction, an aggressive (scores 3&4) invasive front and a poor (scores 3&4) inflammatory infiltrate in HPV-negative HNSCC as compared to the HPV-positive cases **(A)**, and associations between extensive stromal reaction and bigger size tumours, an aggressive invasive front, and a poor inflammatory infiltrate **(B)**.

Including all subsites of HNSCC, HPV-positive cases presented with a non-aggressive tumour invasive front (p<0.001), little desmoplastic stromal reaction (p=0.034), but rich inflammatory host reaction (p<0.001) than the HPV-negative cases (**Figure 1**). Most of the HPV-positive cases presented with a less differentiated (p<0.001, r=0.570), basal like morphology (p<0.001, r=0.363), and had a more pushing (non-aggressive) border type of invasion front (p<0.001, r=-0.538). Consistent with these observations, HPV-positive OPSCC showed non-aggressive tumour invasive front (p=0.001), little desmoplastic stromal reaction (p=0.059, borderline significant), and rich inflammatory host reaction (p=0.013) than HPV-negative OPSCC. In addition, HPV-positive OPSCC displayed a less differentiated (p=0.003, r=0.378), and basal like morphology (p=0.081, r=0.22). Bigger tumours (T3&T4) were associated with a richer desmoplastic stroma (p<0.001, r=0.398), but with a little inflammatory infiltrate (p=0.018, r=0.238). Extensive stromal desmoplasia positively associated with an aggressive tumour invasion front (p<0.001, r=0.426) and presence of metastasis (p=0.045, r=0.203), but inversely associated with the rich inflammatory infiltrate (p<0.001, r=0.-589). The presence of a rich inflammatory infiltrate was negatively associated with an aggressive type of invasive front (p=0.001, r=-0.330) (**Figure 1**).

### Performance of the NGS Method on FFPE Samples and the Effect of Preservation Time

Of special concern was the performance of the targeted NGS panel on aged FFPE material, especially in the FFPE tissue samples preserved in the diagnostics biobank for longer than 10 years, from the 2003-2010 period. The average number of reads per amplicon in the longer time preserved group (2003-2010) was 1709 (n=46) *versus* 1862 (n=58) in the shorter time preserved group (2013-2016), compatible with some lower coverage in the more aged FFPE tissue samples. Of the seven cases that were excluded due to low coverage, five cases were from 2003/04, one case from 2010 and one case form 2015, indicating some concern with respect to performance due to ageing of the FFPE material after more than 5 years preservation time. The excluded cases had number of reads ranging from 20-435 with a median of 159. Targeted sequencing of 101 tumours and 3 tumours from metastatic sites resulted in a total of 2099 single nucleotide variations (SNVs) and 15 indels. No specific difference was, however, noted with respect to SNPs or mutational load between the older samples and the newer ones, stored for 17 and 5 years, respectively.

### Mutational Landscape of HNSCC as Depicted by the Custom Made NGS Panel

**Figure 2:**
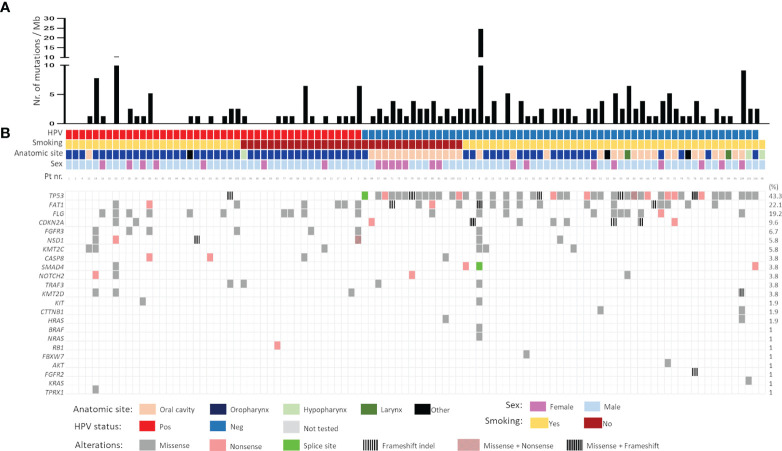
**(A)** Mutational burden and **(B)** mutational spectrum of head and neck squamous cell carcinomas as detected by targeted sequencing. Samples were stratified according to HPV status followed by smoking status. Gene list is shown on the left whereas the corresponding frequency of mutation is shown on the right. Pos- positive; Neg- negative.

The most frequently mutated genes in this Norwegian cohort of HNSCC were *TP53* (n=45, 43.3%), *FAT1* (n=23, 22.1%), *FLG* (n=20, 19.3%), *CDKN2A* (n=10, 9.6%), and *FGFR3* (n=7, 6.7%) (**Figure 2**). The tumour mutational burden (TMB), defined as total number of non-synonymous mutations per coding area of sequenced region, was 2.218 ± 0.2965 for the whole cohort. Smokers displayed higher TMB as compared to non-smokers (2.424 ± 0.4113 and 1.777 ± 0.2940, respectively), although the difference was not statistically significant. TMB was significantly higher in HPV-negative tumours (2.714 ± 0.4292) as compared to HPV-positive (1.515 ± 0.3576, p=0.04). Among the HPV-negative tumours, the highest TMB was detected in oral cavity SCCs (3.239 ± 0.7423), as compared to oropharyngeal SCCs (2.134 ± 0.3041, p=0.2471) (**Figure 2A**). Also, HPV-positive OPSCC had lower TMB (1.526 ± 0.375) as compared to HPV-negative OPSCC (2.133 ± 0.303, p=0.005). There was no difference in TMB in the two different types of sexes, female: (2.1927 ± 0.448) and male: (2.2260 ± 0.357).

The mutational landscape of HPV-positive and HPV-negative tumours was distinct. In HPV-negative carcinomas, mutations were detected mainly in *TP53* (73.3%), *FAT1* (26.7%) and *FLG* (16.7%) whereas in HPV-positive, the common mutations were in *FLG (24.3%) FAT1* (17%) and *FGFR3* (14.6%) genes. A majority of *TP53* mutations were missense (66.7%) followed by nonsense (17.7%) mutations. Frameshift indels (9%) were also frequently observed in the *TP53* gene. Interestingly, 20% (9/44) of the HPV-negative *TP53* mutated tumours had more than one pathogenic *TP53* mutation. Nearly 82% of *FAT1* mutations were missense and only 8% were nonsense, rest being frameshift indels. Inactivating mutations in *FAT1* (n=3) and *CDKN2A* (n=3) were exclusively found in HPV-negative tumours. Contrary to this, genetic alterations in fibroblasts growth factor receptor 3 (*FGFR3*), a potent receptor-tyrosine kinases (RTK) involved in growth and proliferation of cancer cells, were significantly higher in HPV-positive *vs.* HPV-negative tumours (13.95% *vs* 1.73%, p=0.017). All *FGFR3* mutations were missense mutations. Notably the mutations in genes encoding histone methyltransferases, *NSD1* (5.8%) and *KMT2C* (5.8%) were reported, with majority of them being missense. Commonly known mutations in *RAS* (3.9%, combined for *HRAS, NRAS* and *KRAS*), *CASP8* (3.8%), *SMAD4* (3.8%), *NOTCH2* (3.8%), and *TRAF3* (3.8%) were also registered (**Figure 2B**). Focussing only on OPSCC, most mutations in HPV-positive OPSCC were *FLG* (22%) followed by *FAT1* (17.1%), *FGFR3* (14.6%) and *NSD1* (9.8%). Notable mutations in *CASP8* and *KMT2D* (both 7.3%) were also found (**Figure 2B**).

### Mutational Signature of HNSCC

**Figure 3:**
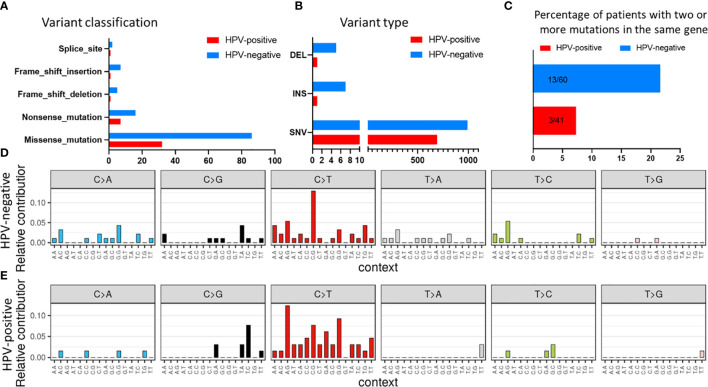
Mutational signature in the HNSCC samples stratified with respect to HPV status for **(A)** variant classification and **(B)** variant type. **(C)** Comparison of number of patients with two or more mutations in the same gene. Mutational profile based on C:G>T:A transitions in **(D)** HPV-negative and **(E)** HPV-positive samples.

SNVs identified in the HNSCC cohort were further evaluated for mutational signatures with respect to presence of HPV and smoking status. The most common class of variant were missense mutations in both HPV-negative and HPV-positive tumours (**Figure 3A**). Also, the frequency of single nucleotide polymorphisms (SNPs) was greater than insertions and deletions both in HPV-positive and HPV-negative cases (**Figure 3B**). Interestingly, more than 21% (13/60) of HPV-negative patients had two mutations in the same gene as compared to only 7.3% (3/41) patients in HPV-positive subtype (**Figure 3C**). As reported before (30), C>T transitions were the most common SNV type in both HPV-negative and HPV-positive tumours, with the frequency being greater in the HPV-positive tumours (**Figures 3D, E**).

**Figure 4:**
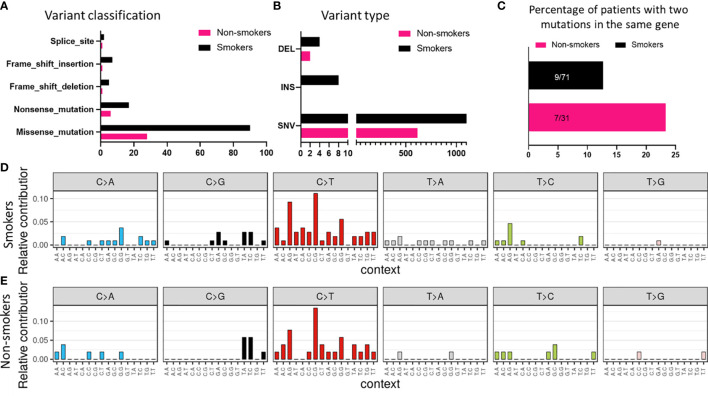
Mutational signature associated with smokers and non-smokers based on **(A)** variant classification, and **(B)** variant type. **(C)** Comparison of number of patients with two or more mutations in the same gene based on smoking status. Mutational profile based on C:G>T:A transitions in **(D)** smokers and **(E)** non-smokers.

Similar observations were made in smokers and non-smokers subtypes with missense mutations being the most common class of variant (**Figure 4A**). Also, the frequency of SNVs was greater than other types of variants in smokers (**Figure 4B**). Surprisingly, the percentage of patients having two mutations in the same gene was more in non-smokers as compared to smokers (**Figure 4C**). Based on the mutational signature, smokers had higher frequency of C>T transitions among different SNVs, as compared to non-smokers (**Figures 4D, E**).

**Figure 5:**
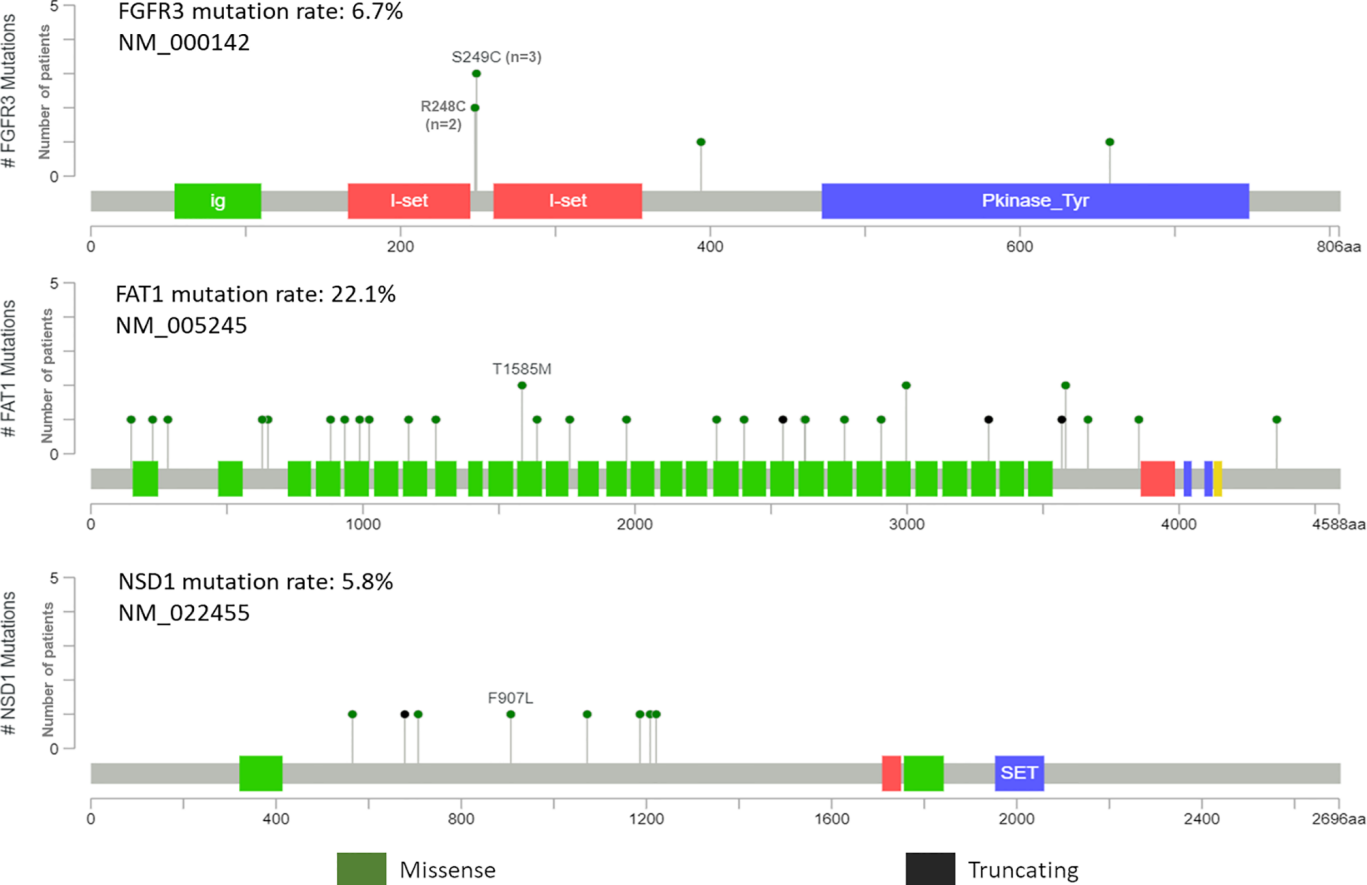
Pictorial representation of single nucleotide variant distribution on domain structure of *FGFR3, FAT1* and *NSD1*.

Potentially targetable mutations in *FGFR3* were identified. Well established canonical missense mutations in R248C and S249C were identified in five patients (4.8%) (**Figure 5**). Surprisingly, all five patients were HPV-positive oropharyngeal SCC with lymph node metastasis. These mutations are used as predictive biomarkers for the use of erdafitinib in patients with locally advanced or metastatic urothelial and bladder carcinomas (NCT02365597). The mutations have been proposed to activate the FGF-receptor *via* ligand-independent dimerization by generating novel cysteine residues (31, 32). Further, different SNVs in proteins coding for *FAT1* and *NSD1* genes are also shown in **Figure 5**. However, the distribution is of *FAT1* and *NSD1* is heterogenous and does not point to any specific pattern.

### Correlations Between Presence of Cancer-Specific Mutations and Clinico-Pathological Parameters

**Figure 6:**
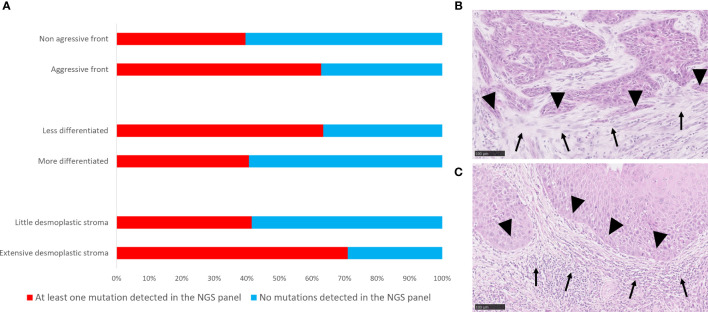
Correlations of mutational landscape with histopathological parameters. **(A)** Bar graph showing distribution of cases with at least one mutation and without mutations in the genes targeted by the NGS panel according to histopathological parameters. **(B)** Histological picture of a tumour with at least one mutation detected by targeted NGS panel, and an extensive stromal desmoplastic response (solid arrows), an aggressive invasive tumour front (solid arrowheads), a less differentiated phenotype and a poor inflammatory response. **(C)** Histological representation of a tumour with no mutations detected by targeted NGS panel and a little stromal desmoplastic response, non-aggressive invasive tumour front (solid arrowheads), a more differentiated phenotype and an intense inflammatory response (solid arrows). Scale bar = 100 μm.

A higher number of cancer-specific mutations were associated with a negative HPV status (p<0.001, r=0.448). No association was observed between the number of SNPs per Mb detected and HPV status or any other clinic-pathological parameters tested. For the whole cohort, presence of at least one cancer-specific mutation was found to be positively associated with an aggressive type of invasive front (p=0.035, r=0.210), and extensive desmoplastic stroma (p=0.019, r=0.234), and negatively associated with the degree of differentiation (p=0.041, r=-203) (**Figure 6**).

### Validation of Correlations Between Mutational Burden and Clinico-Pathological Parameters in TCGA Dataset

**Figure 7:**
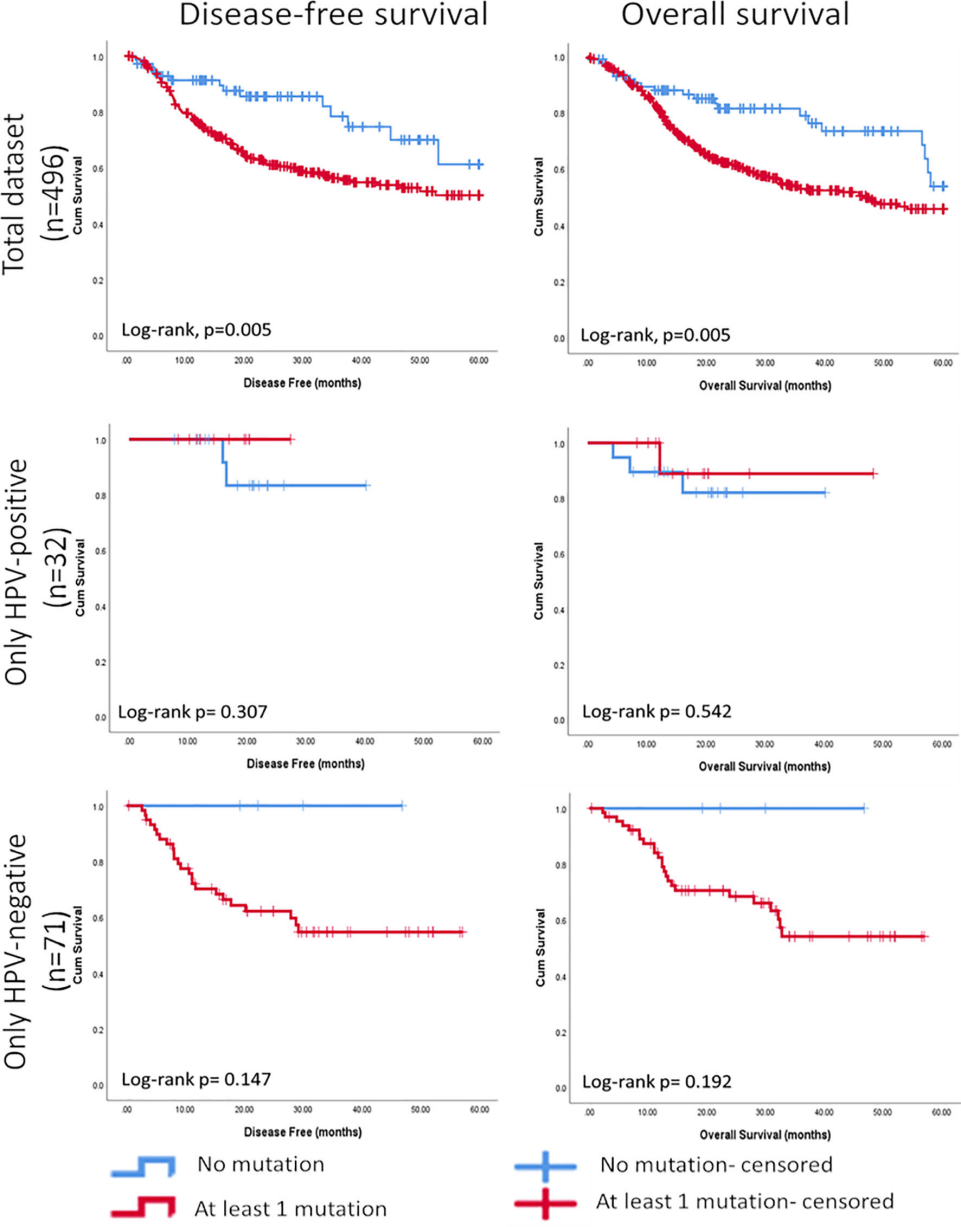
Kaplan-Meier curves showing significant associations between the mutational score based on seven cancer-specific genes found most frequently mutated in HNSCC and further stratified based on HPV-status using the targeted NGS panel and disease-free survival (5 years) and overall survival (5 years).

The Chi Square analysis between mutational score reflecting the presence of at least one mutation in any of the seven most frequently cancer-specific mutated genes identified in our panel (*TP53, FAT1, FGF3, FLG, CDKN2A, KMT2C*, and *NSD1*) and clinicopathological variables available in the TCGA data set for HNSCC showed that the mutation score was significantly associated negatively with differentiation (p<0.001), and positively with perineural invasion (p=0.010), and clinical T-stage (p<0.001). The survival analysis showed that tumours with at least one mutation in one of these genes had shorter 5-year disease-free and overall survival (p=0.005 for both, Log Rank) (**Figure 7**). Further, based on HPV-status stratification, it can be observed that the seven gene panel predicts better disease-free and overall survival in HPV-negative HNSCC (**Figure 7**).

## Discussion

Formalin-fixed paraffin-embedded (FFPE) tumour tissue samples are less optimal for NGS based applications than fresh tissue samples. A major problem is cytosine deamination to uracil, smaller insert sizes, higher duplication rates and an increased frequency of falsely mapped SNVs. Still, Sweiger et al. could show successful low coverage sequencing and copy number detection in FFPE tissue samples stored for more than 18 years (33). Significant improvements were made by Hedegaard and co-workers to improve the output of NGS whole exome sequencing in aged FFPE tissue material, however the successful sequencing was obtained in only 29,5% of the samples (34). They noted in particular an increase in false positive SNVs in FFPE tissue after prolonged storage for more than 3-4 years (34). A similar observation has also been made by others (35). On the other hand, Kerick and co-workers have shown that smaller targeted NGS panels using PCR amplification and an increased coverage will reduce the impact of FFPE induced false positive SNVs in the work up of NGS analysis (36). Our study has used long-term preserved (range 4-17 years) FFPE tumour tissue samples for the NGS analysis. To reduce the impact of fixation, the NGS library was re-amplified before paired-end sequencing on the Illumina MiniSeq platform based on manufacturer’s suggestion to improve number of reads per sample. Also, a robust cut-off of 500 reads for total allelic depth was used to minimize the risk of false positive SNVs due to aged FFPE tumour tissue in our study. We have tested all mutations/annotations involving C-T and A-G conversions in the period 2003-2010 and 2013-2016 without finding significant differences between these time periods. With these limitations kept in mind, the study presents data on the application of a custom-made, HNSCC targeted NGS panel using FFPE material preserved for up to 17 years in a routine diagnostic pathology service. We used the targeted NGS panel to characterize the mutational landscape of a cohort of HNSCC patients with tissues preserved at the Diagnostics Biobank at Department of Pathology, Haukeland University Hospital. Our results are in line with previous studies that found higher TMB in HPV-negative cancers compared to HPV-positive HNSCC (14, 16).

In addition, the panel of genes found most frequently mutated in our study is comparable to the findings from previous studies performed using whole exome sequencing (WES) (14–16). Due to strict cut-off of 500 reads, similar to what is used in diagnostics at HUS, several well reported mutations in the PIK3CA/PTEN pathways were excluded from the final analysis. However, from the remaining detected genes, of interest is our finding of frequent mutations in the gene encoding Nuclear Set Domain Containing Protein 1 (*NSD1*), a histone methyltransferase, in both HPV-positive and negative HNSCC. *NSD1* has been identified as a biomarker for global epigenetic changes in cancer (37, 38) and based on TCGA data, it was recently suggested as a prognostic biomarker in patients with HPV-negative HNSCC (39). Using TCGA data, Bui et al. identified a survival advantage for patients with mutations in the *NSD1* gene. This gene was found altered in approximately 10% of patients with HNSCC, and they proposed that patients with loss-of-function *NSD1* mutations should be considered a distinct clinical subclass of HPV-negative HNSCC, with increased cisplatin sensitivity (39). In addition, we found mutations in *FGFR3* gene, particularly at R248C and S249C that could be used as a targeted therapy especially in HPV-positive HNSCC. Combined together, such findings are usually the result of WES studies and are not prone to be further implemented in the clinical oncology. However, WES is not available at most hospitals owing to its high cost, operational complexity, and long turnover times. On the other hand, lower-cost targeted NGS for cancer specific genes are increasingly affordable and finding its application in clinical oncology. We show here that using a targeted NGS panel is an effective and more affordable way for detecting actionable mutations in archival material preserved for more than 17 years. Moreover, novel mutations unearthed in *FGFR3* in HPV-positive OPSCC justifies the use of NGS based gene panel and suggest valid targets in personalized treatment.

We further wanted to investigate possible association between the presence of mutations in any of the genes investigated and clinical and histopathological parameters. Histopathological assessment of FFPE tissue and surgical resections still remains the cornerstone of diagnosis and are readily available to a pathologist with simple chemical stains. Not only this, but it is well known fact that DNA mutations and their impact on cellular mRNA and protein expression are the main drivers of changes that are evident in the histopathological assessment. As reported in this study, the presence of at least one cancer-specific mutation among the seven most altered genes was associated with less differentiation, an aggressive type of invasive front and a extensive desmoplastic stroma. To our knowledge, this is the first attempt to study and identify associations between mutational load and its stromal host response. In the light of the recent development of immunotherapy, mutational landscape has been previously investigated as a predictor of immune response, but the stromal response has not been investigated in this respect. TMB has been reported as a biomarker for predicting response to immunotherapy in cancer patients (40, 41). This seems to be explained by the thought that tumours with high TMB express a greater diversity of neoantigens, resulting in increased immune recognition when immune checkpoint inhibitors release natural brakes on the immune system. Of note, we did not identify any correlations between TMB or presence of mutations in the investigated genes and presence of a heavier immune infiltrate, but correlations with a more aggressive type of invasion and fibroblastic stromal response were found.

Looking more into the histopathological variables, we have revealed a correlation between presence of at least one cancer specific-mutation and a more desmoplastic response and an aggressive type of invasion at the tumour front but less inflammatory infiltrate. With the new knowledge on the role of cancer associated fibroblasts (CAF) on inducing an immune suppressive phenotype in HNSCC (42) our findings are not surprising. Takahashi et al. found CAFs from HNSCC to express higher B7H1 (PDL1) and B7DC (PDL2) than normal fibroblasts, both putative negative regulators of immune function. Functionally, they were able to suppress T cell proliferation, induce T cell apoptosis and recruit Treg (CD4+Foxp3+) cells. Subpopulations of CAFs with immunosuppressive functions have been recently described in other cancer tissues as well (43). In this respect it is also worth mentioning that our analysis revealed that the HPV-positive cases presented with a non-aggressive tumour invasive front, little desmoplastic stromal reaction, but rich inflammatory host reaction than the HPV-negative cases. This indicates a differential host response to HPV-positive cancers compared to HPV-negative.

The findings of this study were validated on an independent data set. Using TCGA data available for HNSCC, we found that the presence of mutations in at least one of the seven most frequently mutated genes identified by the NGS panel was significantly associated with less differentiation, perineural invasion, and clinical T stage. In addition, we could also perform survival analysis on TCGA data since these data were not available for our HNSCC cohort. The survival analysis showed that tumours with at least one mutation in one of these genes had a shorter 5-year disease-free and overall survival, indicating a clinical relevance for the panel of genes identified by the targeted NGS panel.

## Conclusions

A custom made targeted NGS panel could reliably detect several specific mutations in archival samples of HNSCCs within 17 years of preservation. Using this method, novel associations between mutational burden and clinical and pathological parameters were detected and actionable mutation in HPV-positive HNSCC were discovered. A specific focus was on NGS data obtained on preserved FFPE material, especially with respect to number of PCR amplified reads with a strict cut-off, to uncover more novel and translational mutations.

## Data Availability Statement

The original contributions presented in the study are included in the article/**Supplementary Material**. Further inquiries can be directed to the corresponding author.

## Ethics Statement

The studies involving human participants were reviewed and approved by Regional Committee for Medical and Health Research Ethics (REK Vest, 2011/125). The patients/participants provided their written informed consent to participate in this study.

## Author Contributions

HD, HH, DC, HA, and OV have conceived the study. FE, SM, and HA recruited the patients and collected clinical data. HH and OV performed tissue analysis. SF, SD and RR performed the NGS experiments. HD, HH, DS, OV, and DC analysed data. HD and DC prepared the figures and the manuscript. HA, OV, and DC were supervising the work. All authors contributed to the article and approved the submitted version.

## Funding

This work was supported by The Western Norway Regional Health Authority (Helse Vest Grant No. 911902/2013 and 912260/2019), The Research Council of Norway through its Centres of Excellence funding scheme, (Grant No. 22325), and The Norwegian Centre for International Cooperation in Education (project number CPEA-LT-2016/10106).

## Conflict of Interest

The authors declare that the research was conducted in the absence of any commercial or financial relationships that could be construed as a potential conflict of interest.

## Publisher’s Note

All claims expressed in this article are solely those of the authors and do not necessarily represent those of their affiliated organizations, or those of the publisher, the editors and the reviewers. Any product that may be evaluated in this article, or claim that may be made by its manufacturer, is not guaranteed or endorsed by the publisher.
