# Supplementary material for: Targeted Next-Generation Sequencing of Cancer-Related Genes in a Norwegian Patient Cohort With Head and Neck Squamous Cell Carcinoma Reveals Novel Actionable Mutations and Correlations With Pathological Parameters
Source: Front Oncol. 2021 Sep 24;11:734134. doi: 10.3389/fonc.2021.734134 (PMC8497964; doi:10.3389/fonc.2021.734134)
Supplement: Supplementary file 1 [file Table_1.docx]

| **I: Tumour morphology phenotypes**  ***A****:* ***Degree of keratinization in HNSCCs (%)***  1: 0-5%  2: 5-20%  3: 20-50%  4: >50%  ***B: Fraction maturing cells (%)***  1: <25%  2: 25-50 %  3: 50-75%  4: >75%  ***C: Tumour stromal invasion pattern***  1: None/little: Invasive with mainly pushing borders  2: Some: Invasive with larger tumour strands and nodules  3: Moderate: Invasive with small tumour strands and nodules  4: Extensive: Invasive with smaller tumour strands, islands and single cells | **Score**  1  2  3  4  1  2  3  4  1  2  3  4 |
| --- | --- |
| **II: Tumour host response patterns**  ***D: Inflammatory response***  1: Extensive  2: Moderate  3: Some  4: None/little  ***E: Stromal fibroblastic response (desmoplasia)***  1: None/little  2: Some  3: Moderate  4: Extensive | 1  2  3  4  1  2  3  4 |

Supplementary Table 1. A scoring system for tumour morphology phenotypes and tumour host response patterns in head and neck squamous cell carcinomas (HNSSC).
