# Supplementary material for: Targeted Next-Generation Sequencing of Cancer-Related Genes in a Norwegian Patient Cohort With Head and Neck Squamous Cell Carcinoma Reveals Novel Actionable Mutations and Correlations With Pathological Parameters
Source: Front Oncol. 2021 Sep 24;11:734134. doi: 10.3389/fonc.2021.734134 (PMC8497964; doi:10.3389/fonc.2021.734134)
Supplement: Supplementary file 2 [file Table_2.docx]

| **Gene** | **Exon region** | **Gene** | **Exon region** |
| --- | --- | --- | --- |
| *AKT1* | 2 | *KRAS* | 2-4 |
| *BRAF* | 11, 15 | *KMT2D* | 11,48 |
| *CASP8* | 1-9 | *KMT2C* | 43 |
| *CDKN2A* | 1, 2 | *NF1* | 58 |
| *CTNNB1* | 2 | *NOTCH1* | 28 |
| *CYLD* | 17 | *NOTCH2* | 34 |
| *CUL3* | 8 | *NRAS* | 2-4 |
| *DDR2* | 15 | *NSD1* | 5,6,14,19 |
| *DDX3X* | 6 | *p53* | Full CDS^1^ |
| *FAT1* | Full CDS^1^ | *PTEN* | 1-7, 9 |
| *FBXW7* | 5, 8-11 | *RB1* | 8 |
| *FGFR2* | 7, 9, 12 | *SMAD4* | 8,11 |
| *FGFR3* | 7,9,14,16,18 | *TRAF3* | Full CDS^1^ |
| *FLG* | 3 | *TPRX1* | 2 |
| *HRAS* | 2-4 | *UBR5* | 45 |
| *KIT* | 9,11,13,17,18 |  |  |

^1^CDS: Coding sequence

Supplementary Table 2: Custom-made NGS hot spot gene panel with genes and targeted exon regions.
